# Report on the VIII World Congress of Physchiatry, Athens

**Published:** 1990-03

**Authors:** Susan Johnston


**Report on the VIII World Congress of Physchiatry, Athens, October 1989**

The VIII World Congress of Psychiatry organised by the World Psychiatric Association, took place at the Peace and Friendship Stadium, Athens, Greece from 12th to 19th October, 1989.

The theme of the meeting was "Psychiatry Today—accomplishments and promises". Over 8000 delegates, primarily psychiatrists, from 72 countries and over 2500 trade delegates representing pharmaceutical companies were in attendance.

During seven days almost 3500 papers were presented on every conceivable aspect of psychiatry. Therefore, although only three sessions were specially dedicated to Mental Handicap there was much of relevance in other sessions. I was particularly intrigued by presentations on the neurodevelopmental aspect of schizophrenia, studies and applications of chronobiology and modern genetic techniques. Links were made by the specialists in these fields to the work already done and links with Fragile X and Alzheimer's disease and Down's syndrome. A presentation on the linguistics of psychotic illness and distortion of the "theory of mind" mirrored work done by Professor Fraser in this country with studies of mentally ill, mentally handicapped and mentally ill mentally handicapped people. A fascinating session, particularly for mental handicap work, was, I believe, that on Music, Art and Exercise in which papers were presented demonstrating how behaviour, mental state and cognition could be manipulated by music and exercise. Further, in inaccessible non verbal people emotions could be explored by art and music.

It is impossible to list or describe in a brief report the very many areas of relevance of papers from genetic, biological, psychopharmacological, social or psychotherapeutic approaches and plethora of other fields, suffice to say that we are in a fascinating time of change and knowledge acqusition.

The special sessions on Mental Handicap reviewed the provision of services—both inpatient and community for psychiatrically mentally handicapped in Holland and U.K. The pattern of continuing care for severely mentally handicapped people in and around Paris was described. Consideration was given to incestuous parentage in the aetiology of mental handicap. Psychiatric illness and its forensic implications were presented with remarkably similar findings from centres as varied as Denmark, Holland, U.K. and U.S.A. The need for vigilance in the diagnosis of psychiatric illness especially in mentally handicapped offenders being highlighted with a noticeable accumulation of mildly handicapped offenders in penal institutions, the modern mental handicap service no longer providing for such individuals.

As ever, pervasive developmental disorders were considered but now with new emphasis on neurotransmitter studies and non-invasive brain imaging techniques.

The one presentation by a Stoke Park delegate—JANCAR, J and JOHNSTON, Susan J. "Incest and Mental Handicap"—seemed to be well received and stimulated much discussion out of the session.

I look forward to Stoke Park staff maintaining their contribution to psychiatry in mental handicap at the IX World Congress of Psychiatry in Rio de Janeiro, Brazil, in 1995.

Susan Johnston

